# Legal Responsibility of Consulting Physicians

**Published:** 1874-06

**Authors:** 


*THE WESTERN LANCET—April*, 1874:

LEGAL RESPONSIBILITY OF CONSULTING PHYSICIANS.—The Court instructed the jury as follows:

The law mentions three grades of negligence: slight negligence, which is want of great care and diligence; ordinary negligence, which is the want of ordinary care and diligence; and gross negligence, which is want of even slight care and diligence.

The general rule is that a medical man who attends for a fee is liable for such want of ordinary care and diligence or skill upon his part as leads to the injury of his patient. To render him liable, it is not enough that there has been a less degree of skill than some other medical men might have shown, or a less amount of care than he himself might have bestowed; nor is it enough that he himself acknoweledge some degree of want of care; there must be a want of competent and ordinary care and skill, and to such a degree as to have led to a bad result. But a physician or surgeon is bound, not only to use such skill as he has, but to have a reasonable degree of skill.

If the practitioner frequently informs the patient of his want of skill or the patient is in some other way fully aware of it, the latter cannot complain of the lack of that which he knew did not exist.

The peculiar nature of the services the medical man undertakes to render often makes it his duty to continue them long after he would gladly cease to do so. He may, no doubt, decline absolutely to take charge of a case; but having once begun the task, he cannot abandon it as freely. Even if his services are gratuitous, he must continue them until reasonable time has been given to procure other attendance; and if he is not attending gratuitously, he has no right to desert the patient before the end of the illness which he undertook to treat, without reasonable cause. This is the law as laid down by Sherman and Redfield on Negligence.

There are two questions in this case which you have to pass upon and decide. The first one is whether Dr. Wooster, the defendant treated the case professionally: and if you find that fact in the affirmative, the next inquiry is, did he treat it with that degree of skill which was required of him in his profession of surgeon. The defendant relies upon two grounds of defence. In the first place, he denies that he was employed to treat this case, or that he ever did treat it; and in the second place, he maintains that the treatment of the case was proper and skilful.

You are to take all of the facts and determine these questions from the evidence. If you believe from the evidence that Dr. Todd had exclusive management and control of the case, he alone is responsible for any injury that was done to the plaintiff, if he was injured by malpractice. If you believe that Dr. Todd and the defendant were associated together in business, that they were partners, that they shared the fee and participated in the treatment of this case, the defendant is liable for the acts of Dr. Todd as much as he would be if the acts were his own.

Did the defendant go there simply as a consulting physician? and did he not have, at any time, any control or management of the case? You have heard the testimony upon that point and are to determine from the evidence whether or not he had at any time the management and control of the case, or whether he simply advised Dr. Todd and Dr. Todd accepted and acted upon his advice, or rejected it and acted upon his own advice, at his pleasure.

In regard to the measure of compensation; if you find a verdict in favor of the plaintiff, it is proper for the Court to state that this is not a case in which a jury is authorized to allow what the law calls vindictive or exemplary damages. The law awards such damages in cases of this kind as will compensate the party, as far as compensation can be given by law for an injury which was the necessary unnatural result of this malpractice, if you find there was such. And in fixing that compensation, the law establishes no fixed standard by which the jury may be governed. It is a question which is left to the sound judgment and discretion of the jury. A patient is entitled to receive at the hands of his surgeon such reasonable skill and diligence in the treatment of the case as are ordinarily exercised by well-educated surgeons. And in judging of the degree of skill which the surgeon should bring to the service of his patient, regard is to be had to the advanced state of the profession at the time.

If the jury believe from the evidence that Dr. Todd asked and obtained the consent of the plaintiff to call in the defendant as consulting physician, the defendant is liable for his own acts, if any, of maltreatment, and if his advice and counsel controlled the case, he would be answerable for the character of the treatment.

If the defendant was only a consulting physician without reward, yet if he visited the plaintiff and assisted in the treatment, he is liable for his own acts, if any, of maltreatment.

The jury then retired and after several hours' deliberation returned for further instructions, in reply to the question, "Whether a consulting physician is liable for any damages that may arise from malpractice, whether he gets any compensation or not?" To which the Court replied: "If a consulting physician is called in and he assumes the management of the case and treats the case negligently while he is acting as such consulting physician, he being allowed to take the management of the case by the attending physician, he becomes liable. A physician or surgeon attending gratuitously is liable for gross negligence only. If Dr. Wooster simply acted as consulting physician and Dr. Todd retained the management and control of the case, Dr. Todd alone would be responsible.

"Where a physician receives a fee, a greater degree of care is required of him than in cases where he acts gratuitously. Still the law imposes upon him certain duties and obligations, which he is obliged to perform, although his services may be rendered gratuitously."

